# Comprehensive transcriptomic and metabolomic analysis of porcine intestinal epithelial cells after PDCoV infection

**DOI:** 10.3389/fvets.2024.1359547

**Published:** 2024-05-24

**Authors:** Guangzheng Wang, Yanan Cao, Chao Xu, Shuoshuo Zhang, Yanjie Huang, Shuai Zhang, Wenbin Bao

**Affiliations:** ^1^Key Laboratory for Animal Genetics, Breeding, Reproduction and Molecular Design, College of Animal Science and Technology, Yangzhou University, Yangzhou, China; ^2^Joint International Research Laboratory of Agriculture and Agri-Product Safety, Yangzhou University, Yangzhou, China

**Keywords:** PDCoV, IPEC-J2 cells, transcriptome, metabolome, ferroptosis

## Abstract

**Introduction:**

Porcine deltacoronavirus (PDCoV), an emerging swine enteropathogenic coronavirus with worldwide distribution, mainly infects newborn piglets with severe diarrhea, vomiting, dehydration, and even death, causing huge economic losses to the pig industry. However, the underlying pathogenic mechanisms of PDCoV infection and the effects of PDCoV infection on host transcripts and metabolites remain incompletely understood.

**Methods:**

This study investigated a combined transcriptomic and metabolomic analysis of porcine intestinal epithelial cells (IPEC-J2) following PDCoV infection by LC/MS and RNA-seq techniques. A total of 1,401 differentially expressed genes and 254 differentially accumulated metabolites were detected in the comparison group of PDCoV-infected vs. mock-infected.

**Results and discussion:**

We found that PDCoV infection regulates gene sets associated with multiple signaling pathways, including the neuroactive ligand-receptor interaction, cytokine-cytokine receptor interaction, MAPK signaling pathway, chemokine signaling pathway, ras signaling pathway and so on. Besides, the metabolomic results showed that biosynthesis of cofactors, nucleotide metabolism, protein digestion and absorption, and biosynthesis of amino acid were involved in PDCoV infection. Moreover, integrated transcriptomics and metabolomics analyses revealed the involvement of ferroptosis in PDCoV infection, and exogenous addition of the ferroptosis activator erastin significantly inhibited PDCoV replication. Overall, these unique transcriptional and metabolic reprogramming features may provide a better understanding of PDCoV-infected IPEC-J2 cells and potential targets for antiviral treatment.

## 1 Introduction

Porcine deltacoronavirus (PDCoV) is an emerging porcine enteropathogenic coronavirus. The first reported PDCoV was described in swine rectal swabs from Hong Kong in 2012 (1), and the first outbreak of PDCoV in piglets and sows was announced in Ohio, USA in 2014, followed by rapid spread to other neighboring states in Ohio with a mortality rate of piglets as high as 30%−40% (2). Since then, the emergence of PDCoV has been reported in many countries, including China, Canada, Japan, Korea, Thailand, Lao PDR and Vietnam (3–6). The genome of PDCoV, approximately 25.4 kb, encodes 15 mature nonstructural proteins (nsp2–16), four structural proteins (S protein, E protein, M protein, and N protein) and three accessory proteins (NS6, NS7, and NS7a) (7, 8). PDCoV infection mainly causes intestinal disease in neonatal piglets characterized by acute diarrhea, vomiting and dehydration (9–11). In addition, recent studies have reported that calves, turkeys, and chickens are also susceptible to PDCoV (12, 13). Importantly, PDCoV has the potential to infect humans, posing a significant threat to human and animal health (14). Therefore, studying the infection mechanism of PDCoV is of great importance to prevent the PDCoV epidemic.

Viruses rely on host cell mechanisms to reproduce, generate macromolecules required for their own replication such as amino acids or nucleotide. Viruses assemble by altering the host cell cycle and promoting host's anabolism. PDCoV infection causes changes in the transcriptome of host cells and activates multiple pathways associated with host cell innate immunity (15). PDCoV infection dramatically hampered the expression of type III interferon (IFN-λ) (16), and PDCoV infection also inhibited RIG-I-mediated the production of IFN-β (17). The multiple proteins encoded by PDCoV hijack host innate immunity system to benefit itself replication. Moreover, although apoptosis is thought to be part of the host innate immune system, several viruses promote apoptosis to facilitate the release and dissemination of the viral progeny (18, 19). PDCoV-induced apoptosis was accompanied with the activation of caspase cascades and promoted effective viral replication *in vitro* (20). PDCoV infection induces caspase-dependent apoptosis via intrinsic mitochondrial pathway (20). On the other hand, viruses could regulate the ferroptosis, a form of iron-dependent cell death characterized by accumulated lipid peroxidation. Ferroptosis is reported to be involved in divergent viruses infection processes (21). Activation of ferroptosis have been confirmed to inhibit PEDV replication (22). In contrast, the Infuenza A virus (IAV) infection causes excessive ROS generation in A549 cells, results in oxidative stress and lipid peroxidation, ultimately inducing ferroptosis, which enhance virus replication (23). Therefore, exploring the PDCoV-induced regulatory cell death are beneficial to further investigate the mechanisms of PDCoV infection.

In this study, we scanned the differentially expressed genes and metabolites in PDCoV infected IPEC-J2 cells at 24 h using transcriptomic and metabolomic approaches. The combined transcriptomic and metabolomic analysis showed that multiple signaling pathways are involved in PDCoV infection, such as PI3K/AKT signaling pathway and ferroptosis. Of note, erastin-induced ferroptosis impeded PDCoV replication. Overall, PDCoV infection alters the transcription and metabolism of host cells to promote its replication.

## 2 Materials and methods

### 2.1 Cell culture

IPEC-J2 cells used in this study were kept in our laboratory. The IPEC-J2 cells were cultured in DMEM medium supplemented with 10% fetal bovine serum (FBS, GIBCO, Australia), and 1% Pen-Strep [penicillin (100 U/ml), streptomycin (0.1 mg/ml); Solarbio, Beijing, China]. Cells were cultured at 37°C in a humidified incubator set at 5% CO_2_.

### 2.2 PDCoV infection

The IPEC-J2 cells were seeded in 100 mm cell culture dishes and cultured overnight in an incubator at 37°C with 5% CO_2_. PDCoV at a 1 multiplicity of infection (MOI) was used for adsorption in monolayer of IPEC-J2 cells for 1 h at 37°C with 5% CO_2_, then the inoculum was removed and washed three times with phosphate-buffered saline (PBS), DMEM supplemented with 2% FBS and 1% pancreatin was subsequently added, and incubation was continued at 37°C with 5% CO_2_ for 24 h.

### 2.3 Sample collection

The cells were collected and washed with ice-cold PBS three times. For metabolomic analysis, 500 μl ice-cold methanol-water (4:1, V/V, MACKLIN, Shanghai, China) was added to scrape off the cells, then transferred the collected cells to a 1.5 ml centrifuge tube, and added another 500 μl ice-cold methanol-water (4:1, V/V) and stored in −80°C; For transcriptomic analysis, cells were collected, and total RNA was extracted by RNAiso Plus (TaKaRa, Dalian, China) according to the manufacturer's instructions and the extracted RNA was stored in −80°C.

### 2.4 Transcriptome sequencing and analysis

Transcriptome sequencing and differential expression analysis were provided by Novogene (Beijing, China). Genes with adjust *P* ≤ 0.05 and |log_2_FC| ≥ 1 were considered as differentially expressed genes (DEGs). Function classification and enrichment analysis of DEGs were performed using GOseq software based on GO database (http://geneontology.org/page/go-enrichment-analysis). Based on the annotations of DEGs, transcripts were classified into three categories, including biological process (BP), cellular component (CC) and molecular function (MF). Pathway enrichment was analyzed based on the KEGG database (https://www.kegg.jp/kegg/kegg1.html) and carried out using the KOBAS 3.0 web server (with a *P* ≤ 0.05) at each comparison level for different genotypes and treatments.

### 2.5 Metabolites analysis

Extraction of metabolites and LC-MS/MS analysis were provided by Novogene (Beijing, China). The identified differential metabolites were combined by the VIP value of the PLS-DA model (≥1), the *P*-value of student's *t*-test (≤0.05) and |log_2_FC| (≥0.5). The eligible metabolites were considered as significantly changed. The KEGG database was used to annotate and display the differential metabolites. Other analyses included partial least-squares discriminant analysis (PLS-DA) and pathway enrichment.

### 2.6 Quantitative real-time PCR

Relative expression levels of selected genes were examined by RT-qPCR using AceQ Universal SYBR qPCR Master Mix (Vazyme, Nanjing, China). The relative gene expression levels of the 8 genes were normalized to *GAPDH* expression used the 2^−ΔΔCT^ method. The primers used in RT-qPCR were listed in Table 1. The RT-qPCR reactions were performed on the StepOnePlus Real-Time PCR System (Thermo Fisher Scientific, Massachusetts, USA).

**Table 1:** Primers for RT-qPCR.

**Primers**	**Sequences**	**Product length**
*GAPDH*	F: ACATCATCCCTGCTTCTACTGG	188
	R: CTCGGACGCCTGCTTCAC	
*TFRC*	F: GCTTTGAAGAACCAGATCGC	773
	R: AGCACGGAAGAAGTCTCCAC	
*ACSL5*	F: GACTGGACATCAGGCCATGT	654
	R: ACTGTCGATTTGAGTGCGAA	
*ANXA2*	F: TTTGACCAACCGCAGCAATG	83
	R: GCGCTGATGCAAGTTCCTTT	
*PSAP*	F: GACAATGCCACTGAGCAGGA	583
	R: ATCCAACCAGCCCACAGATG	
*VNN2*	F: GGAGTATTGGCAGGTATGCAC	235
	R: GACAGGCTCAGATGTTCCGT	
*ENPP*	F: CCTGTGACCCTTCAATTTTGCC	148
	R: TGGTAAAGCAGACAGACGGT	
*IRF1*	F: AAGCATGGCTGGGACATCAA	75
	R: TGCTTTGTATCGGCCTGTGT	

### 2.7 Western blotting assays

The cells were washed three times with PBS and lysed with RIPA Cell Lysis Buffer, and then equal amounts of protein were subjected to sodium dodecyl sulfate polyacrylamide gel electrophoresis (SDS–PAGE). The samples were transferred to PVDF membranes, and the membranes were next blocked with 5% skim milk powder dissolved in TBST (TBS containing 2% Tween 20) for 2 h at room temperature. The membranes were then incubated with specific primary antibodies for 12 h, washed three times with TBST, and incubated with secondary antibodies for 2 h. The bands were imaged using a Tanon-5200 Chemiluminescent Imaging System (Tanon Science & Technology Co., Ltd.).

### 2.8 Statistical analysis

Statistical analysis was performed by Statistical Program for Social Sciences (SPSS 16.0, SPSS Inc., Chicago, IL, USA). Data were presented as the mean ± standard deviation (SD), and differences between control and experimental groups employed Student's *t*-test and one-way analysis of variance (ANOVA). For all analysis, *P*-values of < 0.05 were considered statistically significant (^*^P < 0.05; ^**^P < 0.01).

## 3 Results

### 3.1 PDCoV infection induces the changes of transcriptome profile in IPEC-J2 cells

**Figure 1:**
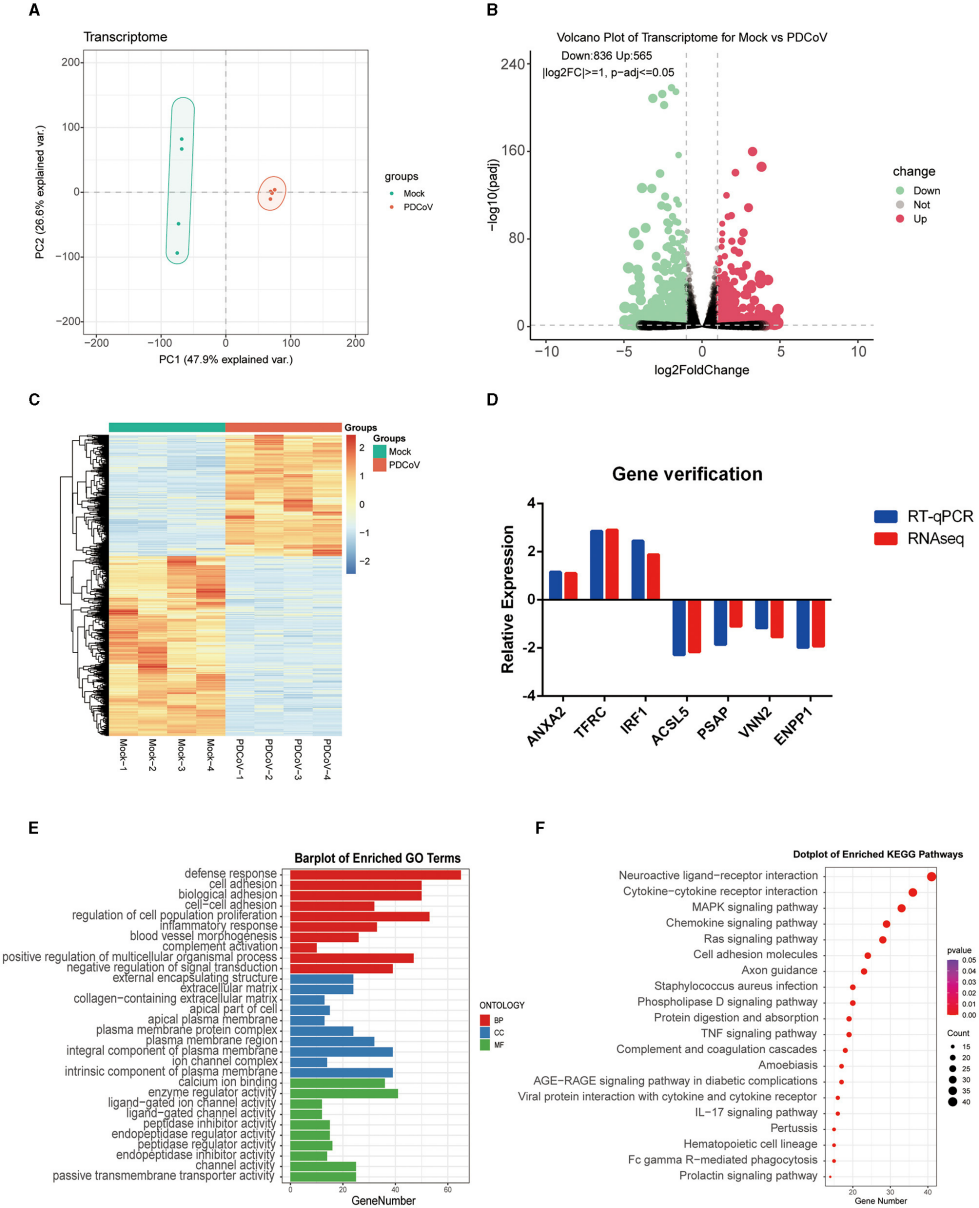
Analysis of transcriptome changes in the IPEC-J2 cells during PDCoV infection. **(A)** Principal component analysis (PCA). **(B)** Volcano plot of differentially expressed genes between PDCoV and Mock group. Each plot represents a gene identified. **(C)** Heatmap of differentially expressed genes. **(D)** Gene verification. Differentially expressed genes levels were detected by RT-qPCR. The levels of selected genes were normalized with GAPDH. **(E)** Top 10 significantly enriched GO terms of each category, including biological process (BP), cellular component (CC), and molecular function (MF). **(F)** KEGG pathway classification of differentially expressed genes in PDCoV infected cells (MOI = 1).

To determine the transcriptome changes in the peak of PDCoV replication, RNA sequencing was pursued in PDCoV infected IPEC-J2 cells at 24 h. Principal component analysis (PCA) showed that the samples of PDCoV-infected group and mock group were well separated (Figure 1A), indicating that the gene expression pattern of PDCoV-infected cells was differ from mock-infected cells.

A total of 1,401 DEGs were detected, of which 565 DEGs were upregulated and 836 DEGs were downregulated. All DEGs identified were mapped to the volcano map (Figure 1B). Gene expression profiles with significant difference was presented in the heat map (Figure 1C). To further validate the accuracy of transcriptome data, we randomly selected *ANXA2, TFRC* and *TIRF1* from upregulated DEGs, *PSAP*, AC*SL5C3, VNN2*, and *ENPP* from downregulated DEGs for RT-qPCR analysis. Consistent with the RNA sequencing data, the mRNA levels of *ANXA2, TFRC*, and *TIRF1* were significantly increased and the mRNA levels of *PSAP*, AC*SL5C3, VNN2*, and *ENPP* were significantly decreased, indicating the reliability of the RNA sequencing results (Figure 1D).

All DEGs were annotated to the Gene Ontology (GO) database (http://geneontology.org), and enriched to 528 signaling pathways. The top 10 pathways with the highest enrichment in each category were selected for display (Figure 1E). GO analysis in BP was mainly related with defense response, cell adhesion, and biological adhesion; CC was mainly enriched in external encapsulating structure, extracellular matrix and collagen-containing extracellular matrix; MF was mainly enriched in calcium ion binding, enzyme regulator activity and ligand–gated ion channel activity. Furthermore, 1,401 DEGs were annotated to the Kyoto Encyclopedia of Genes and Genomes (KEGG) database (http://www.kegg.jp/kegg/pathway.html), and assigned to 68 different KEGG pathways. The KEGG bubble plots show the top 20 enriched signaling pathways (Figure 1F), relating to neuroactive ligand–receptor interaction, cytokine-cytokine receptor interaction, MAPK signaling pathway, chemokine signaling pathway and ras signaling pathway.

### 3.2 Metabolomic changes in PDCoV infected IPEC-J2 cells

**Figure 2:**
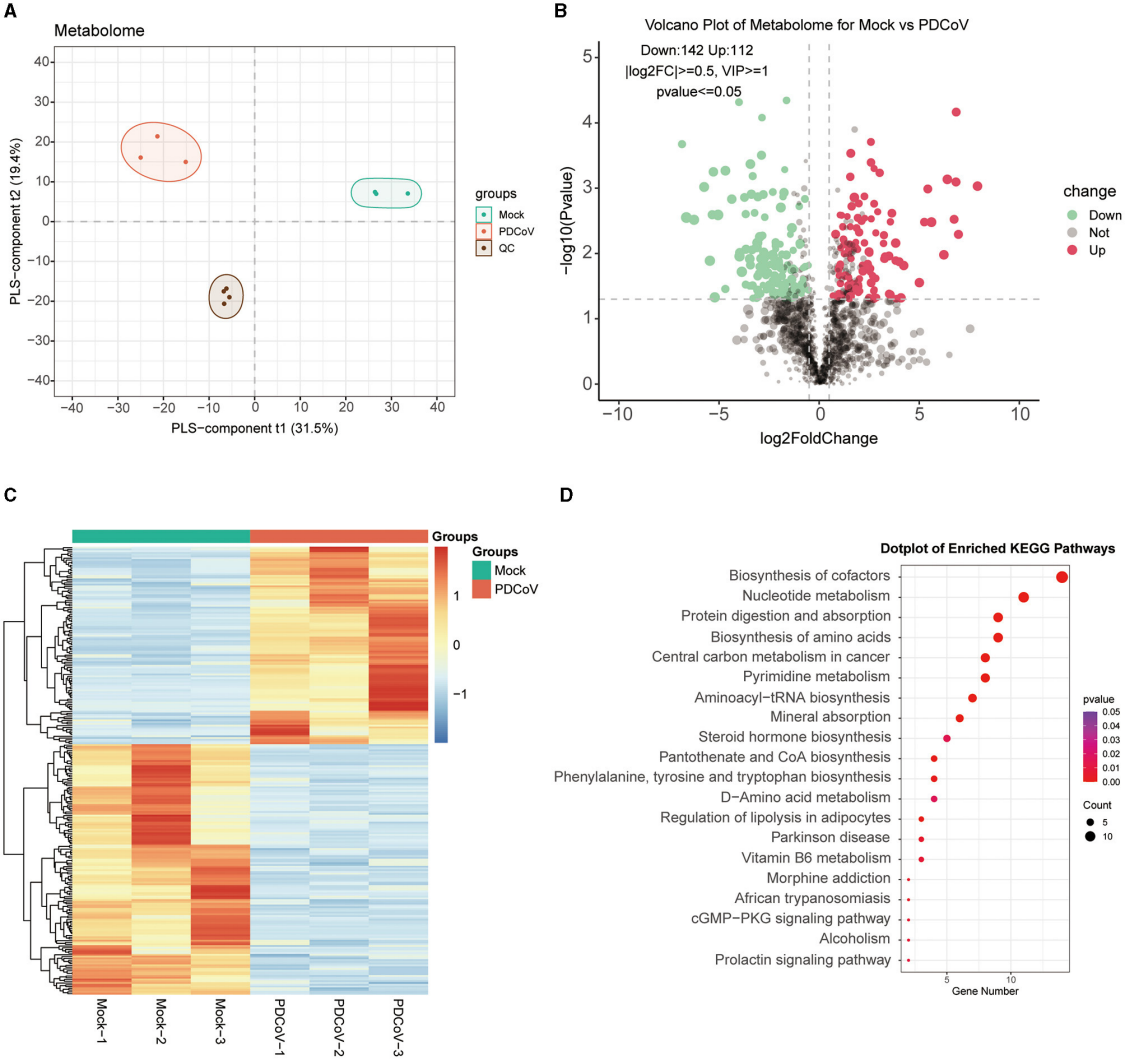
Differential metabolite identification and pathway enrichment analysis. **(A)** PLS-DA model. **(B)** Volcano plot of differentially expressed metabolites between PDCoV and mock group. Each plot represents a metabolite identified. **(C)** Heatmap analysis of significantly changed metabolites between PDCoV and mock group. **(D)** KEGG pathway classification of differentially expressed metabolites upon PDCoV infection (MOI = 1).

To further investigate the different metabolites involved in the cellular responses to PDCoV infection, metabolomic changes were carried out on PDCoV infected IPEC-J2 cells by LC/MS. The PDCoV-infected and mock groups were easily distinguished, and the PLS-DA model was reliable with a large gap and good intra-group clustering within groups, indicating that PDCoV infection caused the metabolite changes (Figure 2A). All identified metabolites were plotted as volcano maps (Figure 2B), and 254 differential metabolites were identified in the PDCoV-infected group compared to the mock group, in which red dots represented upregulated 112 metabolites and blue dots represented downregulated 142 metabolites. The top 10 upregulated metabolites upon PDCoV infection were Methionine, Pidotimod, PG(22:5/22:5), L-Valine, DL-α-Aminocaprylicacid, 5-Aminopentanoate, Cuminaldehyde, N-Acetyl-aspartic acid, L-(+)-Citrulline, N-Acetylaspartic acid. Meanwhile, the top 10 downregulated metabolites were LPG 20:1, N1-methyl-5-methoxy-2-({2-[(methylamino)carbonyl]phenyl}thio) benzamide, Prostaglandin H2, Penicillin G, DL-Carnitine, 6-methyl-4-(morpholinomethyl)-2H-chromen-2-one, N1-pyrazin-2-yl-4chlorobenzamide, L-Glutathione (reduced), D-Mannose 6-phosphate, DL-Arginine. Additionally, Hierarchical clustering was further displayed to show the expression patterns of the differential metabolites between and within the PDCoV and mock group (Figure 2C).

Subsequently, differential expressed metabolites were enriched based on the KEGG database (http://www.kegg.jp/kegg/pathway.html), the bubble map lists the top 20 most enriched KEGG pathways (Figure 2D), which specifically contained Biosynthesis of cofactors, Nucleotide metabolism, Protein digestion and absorption, Biosynthesis of amino acids and Central carbon metabolism in cancer. Notably, the expression of various amino acids markedly changed. A significant upregulation of L-glutamine and a significant downregulation of gamma-Glutamylcysteine indicated that PDCoV infection resulted in glutamine accumulation. Furthermore, we also found that PDCoV infection altered the nucleotide metabolism of host cells, with significant changes in the expression of various nucleotides essential for cell proliferation and viral replication. The nucleotides may be consumed by PDCoV for its own replication. In addition, the expression of 5′-Adenylic acid was significantly downregulated, which also suggested the consumption of nucleotides of host cells upon PDCoV infection. Overall, these results suggested that PDCoV utilize numerous host components like amino acids and nucleotides to complete their life cycle.

### 3.3 Integrated analysis of transcriptome and metabolome data

**Figure 3:**
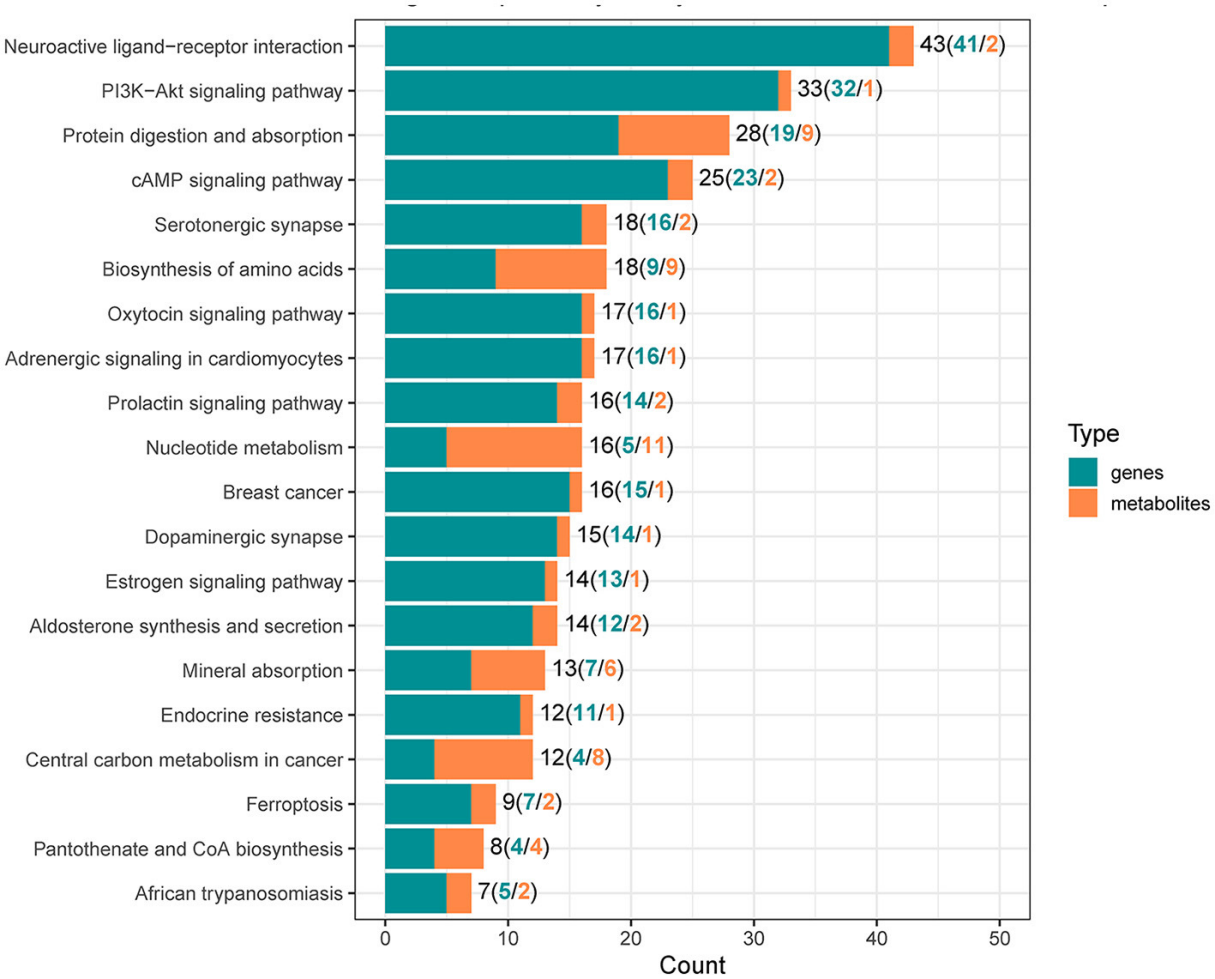
Integrated analysis of the differentially expressed genes and differentially accumulated metabolites.

**Figure 4:**
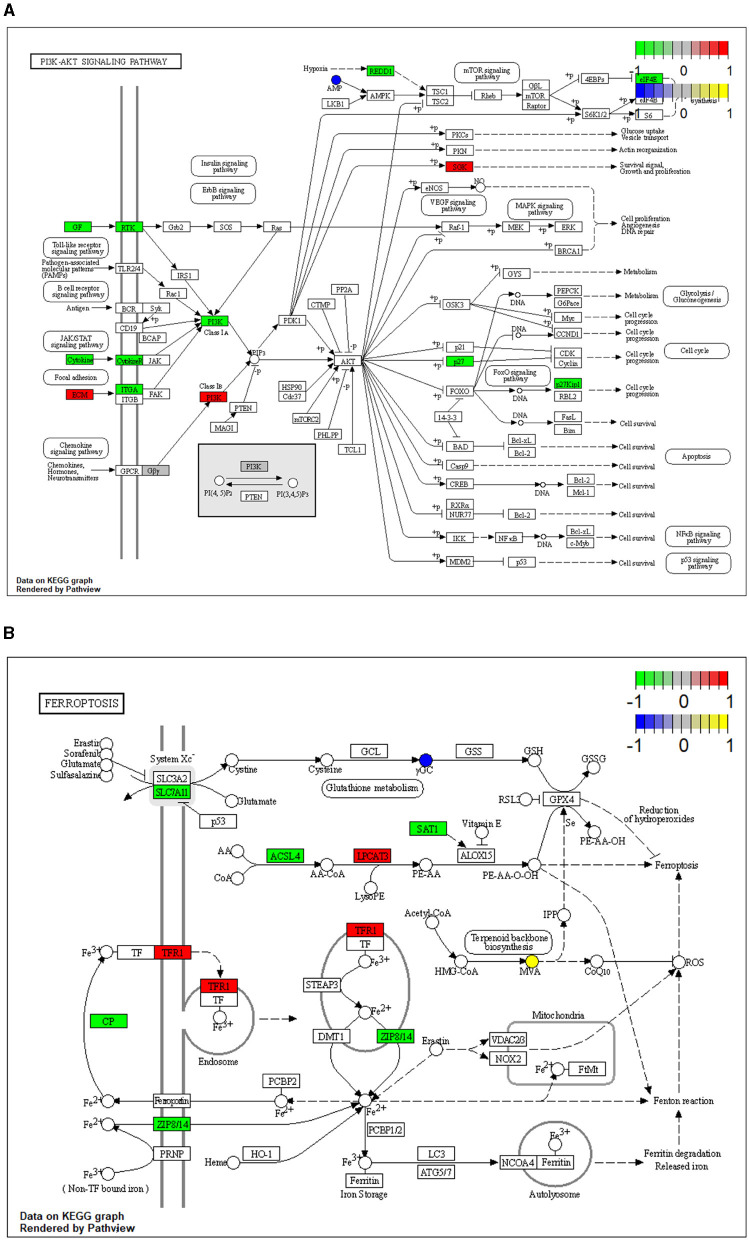
Mapping of differentially expressed genes (DEGs) and differentially accumulated metabolites (DAMs) to KEGG pathways. **(A)** PI3K/Akt signaling pathway (ssc04151). **(B)** Ferroptosis (ssc04216). The squares represent genes and the circles represent metabolites. Green indicates gene downregulation, red indicates gene upregulation, blue represents metabolite downregulation, and yellow represents metabolite upregulation.

To reveal the molecules and signaling pathways associated with the response to PDCoV infection, we combined analysis of the transcriptome and metabolome between the mock and PDCoV groups. DEGs and differentially accumulated metabolites (DAMs) caused by PDCoV infection were mapped onto the KEGG pathway map. DEGs and DAMs were enriched to PI3K-Akt signaling pathway (*P* < 0.01) and ferroptosis signaling pathway (*P* < 0.01; Figure 3). Specifically, 32 key genes and one metabolite (5′-Adenylic acid) were mapped to the PI3K/Akt signaling pathway. In addition, seven differentially expressed key genes (*ACSL5, TFRC, SLC39A8, LPCAT3, SLC7A11, SAT2*, and *CP*) and two DAMs (Mevalonic acid, gamma-Glutamylcystein) were participated in ferroptosis signaling pathway (Figure 4).

### 3.4 Activator of ferroptosis signaling pathway inhibits PDCoV infection

**Figure 5:**
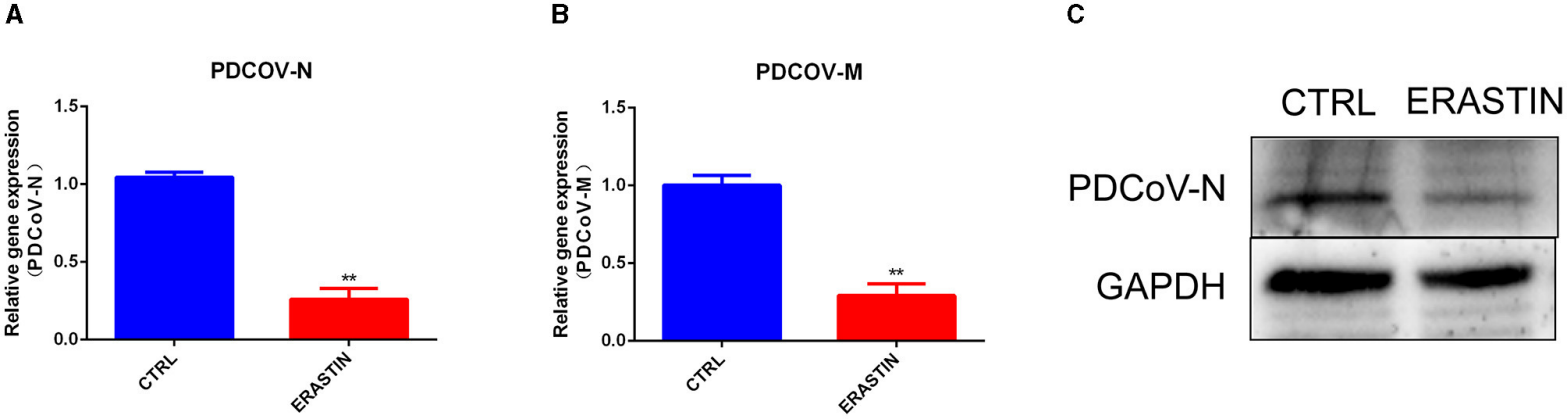
Erastin inhibits PDCoV replication in IPEC-J2 cells. The PDCoV-*N* mRNA level **(A)** and PDCoV-*M* mRNA level **(B)** were detected by RT-qPCR (MOI = 1). **(C)** The PDCoV-N protein level was detected by western blotting (MOI = 1). **P < 0.01.

Ferroptosis is an important cellular non-apoptotic form of regulated cell death that plays a role in several viral infections (23, 24). To further explore the specific role of ferroptosis pathway plays in PDCoV infection, we used erastin, a classical activator of ferroptosis (22), to pretreated with IPEC-J2 cells, and then infected with PDCoV at an MOI of 1. RT-qPCR (Figures 5A, B) and western blotting (Figure 5C) results indicated that erastin treatment hampered the transcription of viral genes and the expression of viral protein.

## 4 Discussion

Since PDCoV was first identified in 2012 and led to an outbreak of porcine diarrhea disease in the USA in 2014, it had quickly become a new threat to the swine industry. To date, transcriptomics has been extensively used to unravel the mechanisms of viral infection and to find the key genes and signaling pathways involved in viral infection (25). Moreover, metabolomics has also been utilized to investigate the effects of viral infection on host metabolism and to screen possible therapeutic targets in metabolic networks (26). In this study, we investigated the effects of PDCoV infection on host transcripts and metabolites based on a combined transcriptomic and metabolomic analysis. At the metabolic level, PDCoV infection mainly altered the metabolism of nucleotides, proteins, and amino acids. At the transcriptional level, we found that PDCoV infection activated PI3K/Akt signaling pathway and ferroptosis signaling pathway, altered multiple related genes including *TFRC, ACSL5, SLC39A8, LPCAT3, SLC7A11, SAT2*, and *CP*, and the activation of ferroptosis signaling pathway significantly inhibited PDCoV replication.

Amino acids are the most important substances in living organisms. Not only they are used in the synthesis of proteins and other important biomolecules, but they also provide intermediate metabolites for the tricarboxylic acid cycle and gluconeogenesis. Amino acids metabolism is involved in the process of viral infection and innate immunity (27). In the present study, PDCoV infection resulted in changes in the expression patterns of a variety of amino acids and nucleotides. L-glutamine, methionine, L-tryptophan, phenylalanine, L-tyrosine, L-aspartate, L-valine, citrulline and L-kynurenine were obviously upregulated, while L-cysteine and its derivatives showed a significant downregulation. As intracellular parasites, viruses completely reply on cell energy and molecular machinery to enter, proliferation, and build successful infection. In general, viruses are capable of re-shaping host metabolic network during their infection process (28, 29). It is reported that African swine fever virus hijack host energy and animo acid metabolism to promote viral infection (30). KSHV induces alterations in the glycolytic pathway by enhancing glutamine uptake, which provides intermediates for the tricarboxylic acid cycle (31, 32). Hence, we hypothesize that, agreement with KSHV, PDCoV upregulates the expression of various amino acids, especially glutamine, increases the intracellular amino acid pool and regulates the tricarboxylic acid cycle in the host cell to provide amino acids and energy for its own replication. Viruses meet the demands of viral nucleic acid synthesis by regulating nucleotide anabolism (33). Elevated levels of UDP, CMP, cytosine, uracil, UMP and GMP during NDV infection. Alterations in nucleotide metabolism may play an important role in promoting rapid replication of the NDV virus genome. CSFV-infected PK-15 cells showed decreased inosine, inosinic acid and guanine content, suggesting CSFV infection promotes nucleobase depletion by viral RNA synthesis activity (34). In our study, the production of uracil, adenine, thymine, cytosine and most of their derivatives or raw materials were significantly reduced, which may due to the high consumption of nucleotides during the rapid replication of PDCoV in the early stages. These results reveal that PDCoV regulate the primary metabolite (i.e., Biosynthesis of amino acids and Nucleotide metabolism) to promote its replication.

Ferroptosis is an iron-dependent, non-apoptotic form of regulated cell death (RCD). It is mainly mediated by iron-dependent lipid peroxidation and is characterized by elevated intracellular levels of iron and phospholipid peroxides (35), with excess iron promoting abnormal accumulation of ROS and accelerating lipid peroxidation (36). In our study, transcriptomic results showed that the expression of several differentially significant genes was associated with the ferroptosis pathway. In specifically. activating ferroptosis signaling pathway by erastin significantly impeded PDCoV infection. Besides, it was found that SIV virus upregulated the expression of TFRC during the infection process, causing the cells to take in large amounts of iron before the initiation of defense mechanisms, which eventually induced ferroptosis in the cells (23). Moreover, metabolomic results also showed that γ-GC content was significantly downregulated. γ-GC functions as synthesizing and upregulating GSH, which is a reduced substrate for GPX4, an important negative regulator of ferroptosis. It was shown that γ-GC significantly inhibited ferroptosis in neuronal cells (37). Our results suggest that PDCoV infection activates the ferroptosis signaling pathway in IPEC-J2 cells, and the host use the activation of ferropotosis to inhibit the virus replication.

## Data availability statement

The original contributions presented in the study are publicly available. This data can be found at: https://www.ncbi.nlm.nih.gov/bioproject/, PRJNA1078001; https://www.ebi.ac.uk/metabolights/, MTBLS9591.

## Author contributions

GW: Writing – original draft, Data curation, Formal analysis, Investigation, Methodology, Validation. YC: Investigation, Software, Writing – review & editing. CX: Data curation, Methodology, Software, Writing – review & editing. ShuoZ: Methodology, Visualization, Writing – review & editing. YH: Formal analysis, Software, Writing – review & editing. ShuaZ: Conceptualization, Funding acquisition, Project administration, Resources, Supervision, Validation, Writing – review & editing. WB: Writing – review & editing, Funding acquisition, Project administration, Resources, Validation.

## References

[1] Woo PCY Lau SKP Lam CSF Lau CCY Tsang AKL Lau JHN. Discovery of seven novel mammalian and avian coronaviruses in the genus deltacoronavirus supports bat coronaviruses as the gene source of alphacoronavirus and betacoronavirus and avian coronaviruses as the gene source of gammacoronavirus and deltacoronavirus. J Virol. (2012) 86:3995–4008. 10.1128/JVI.06540-11 22278237 PMC3302495

[2] Wang L Byrum B Zhang Y. Detection and genetic characterization of deltacoronavirus in pigs, Ohio, USA, 2014. Emerg Infect Dis. (2014) 20:140296. 10.3201/eid2007.140296 24964136 PMC4073853

[3] Wang Y-W Yue H Fang W Huang Y-W. Complete genome sequence of porcine deltacoronavirus strain CH/Sichuan/S27/2012 from Mainland China. Genome Announc. (2015) 3:e00945-15. 10.1128/genomeA.00945-15 26337879 PMC4559728

[4] Ajayi T Dara R Misener M Pasma T Moser L Poljak Z. Herd-level prevalence and incidence of porcine epidemic diarrhoea virus (PEDV) and porcine deltacoronavirus (PDCoV) in swine herds in Ontario, Canada. Transbound Emerg Dis. (2018) 65:1197–207. 10.1111/tbed.12858 29607611 PMC7169835

[5] Lee S Lee C. Complete genome characterization of korean porcine deltacoronavirus strain KOR/KNU14-04/2014. Genome Announc. (2014) 2:e01191-14. 10.1128/genomeA.01191-14 25428966 PMC4246158

[6] Stramer SL Moritz ED Foster GA Ong E Linnen JM Hogema BM. Hepatitis E virus: seroprevalence and frequency of viral RNA detection among US blood donors. Transfusion. (2016) 56:481–8. 10.1111/trf.13355 26434952

[7] Fang P Fang L Liu X Hong Y Wang Y Dong N. Identification and subcellular localization of porcine deltacoronavirus accessory protein NS6. Virology. (2016) 499:170–7. 10.1016/j.virol.2016.09.015 27661736 PMC7111631

[8] Fang P Fang L Hong Y Liu X Dong N Ma P. Discovery of a novel accessory protein NS7a encoded by porcine deltacoronavirus. J Gen Virol. (2017) 98:173–8. 10.1099/jgv.0.000690 27995863 PMC7079566

[9] Chen Q Gauger P Stafne M Thomas J Arruda P Burrough E. Pathogenicity and pathogenesis of a United States porcine deltacoronavirus cell culture isolate in 5-day-old neonatal piglets. Virology. (2015) 482:51–9. 10.1016/j.virol.2015.03.024 25817405 PMC7111688

[10] Jung K Hu H Saif LJ. Porcine deltacoronavirus infection: etiology, cell culture for virus isolation and propagation, molecular epidemiology and pathogenesis. Virus Res. (2016) 226:50–9. 10.1016/j.virusres.2016.04.009 27086031 PMC7114557

[11] Zhang J. Porcine deltacoronavirus: overview of infection dynamics, diagnostic methods, prevalence and genetic evolution. Virus Res. (2016) 226:71–84. 10.1016/j.virusres.2016.05.028 27270129 PMC7114555

[12] Boley PA Alhamo MA Lossie G Yadav KK Vasquez-Lee M Saif LJ. Porcine deltacoronavirus infection and transmission in Poultry, United States1. Emerg Infect Dis. (2020) 26:255–65. 10.3201/eid2602.190346 31961296 PMC6986833

[13] Jung K Hu H Saif LJ. Calves are susceptible to infection with the newly emerged porcine deltacoronavirus, but not with the swine enteric alphacoronavirus, porcine epidemic diarrhea virus. Arch Virol. (2017) 162:2357–62. 10.1007/s00705-017-3351-z 28374120 PMC7086908

[14] Li W Hulswit RJG Kenney SP Widjaja I Jung K Alhamo MA. Broad receptor engagement of an emerging global coronavirus may potentiate its diverse cross-species transmissibility. Proc Natl Acad Sci USA. (2018) 115:E5135–43. 10.1073/pnas.1802879115 29760102 PMC5984533

[15] Gladue D Jiang S Li F Li X Wang L Zhang L. Transcriptome analysis of PK-15 cells in innate immune response to porcine deltacoronavirus infection. Plos ONE. (2019) 14:e0223177. 10.1371/journal.pone.0223177 31574122 PMC6773216

[16] Liu S Fang P Ke W Wang J Wang X Xiao S. Porcine deltacoronavirus (PDCoV) infection antagonizes interferon-λ1 production. Vet Microbiol. (2020) 247:108785. 10.1016/j.vetmic.2020.108785 32768229 PMC7331541

[17] Luo J Fang L Dong N Fang P Ding Z Wang D. Porcine deltacoronavirus (PDCoV) infection suppresses RIG-I-mediated interferon-β production. Virology. (2016) 495:10–7. 10.1016/j.virol.2016.04.025 27152478 PMC7111668

[18] Zhang H Li F Pan Z Wu Z Wang Y Cui Y. Activation of PI3K/Akt pathway limits JNK-mediated apoptosis during EV71 infection. Virus Res. (2014) 192:74–84. 10.1016/j.virusres.2014.07.026 25116390

[19] Ren Z Yu Y Zhang X Wang Q Deng J Chen C. Exploration of PDCoV-induced apoptosis through mitochondrial dynamics imbalance and the antagonistic effect of SeNPs. Front Immunol. (2022) 13:972499. 10.3389/fimmu.2022.972499 36081520 PMC9446457

[20] Lee YJ Lee C. Porcine deltacoronavirus induces caspase-dependent apoptosis through activation of the cytochrome c-mediated intrinsic mitochondrial pathway. Virus Res. (2018) 253:112–23. 10.1016/j.virusres.2018.06.008 29940190 PMC7114866

[21] Gao J Wang Q Tang Y-D Zhai J Hu W Zheng C. When ferroptosis meets pathogenic infections. Trends Microbiol. (2023) 31:468–79. 10.1016/j.tim.2022.11.006 36496309

[22] Zhang H Li Y Yang R Xiao L Dong S Lin J. Erastin inhibits porcine epidemic diarrhea virus replication in Vero cells. Front Cell Infect Microbiol. (2023) 13:1142173. 10.3389/fcimb.2023.1142173 36936772 PMC10015705

[23] Cheng J Tao J Li B Shi Y Liu H. Swine influenza virus triggers ferroptosis in A549 cells to enhance virus replication. Virol J. (2022) 19:104. 10.1186/s12985-022-01825-y 35715835 PMC9205082

[24] Li Y Bao Y Li Y Duan X Dong S Lin J. RSL3 inhibits porcine epidemic diarrhea virus replication by activating ferroptosis. Viruses. (2023) 15:2080. 10.3390/v15102080 37896857 PMC10612067

[25] Liu X Zhu L Liao S Xu Z Zhou Y. The porcine microRNA transcriptome response to transmissible gastroenteritis virus infection. PLoS ONE. (2015) 10:e0120377. 10.1371/journal.pone.0120377 25781021 PMC4363316

[26] Manchester M Anand A. Metabolomics: strategies to define the role of metabolism in virus infection and pathogenesis. Adv Virus Res. (2017) 98:57–81. 10.1016/bs.aivir.2017.02.001 28433052

[27] Tome D. Amino acid metabolism and signalling pathways: potential targets in the control of infection and immunity. Eur J Clin Nutr. (2021) 75:1319–27. 10.1038/s41430-021-00943-0 34163018 PMC8220356

[28] Moreno-Altamirano MMB Kolstoe SE Sánchez-García FJ. Virus control of cell metabolism for replication and evasion of host immune responses. Front Cell Infect Microbiol. (2019) 9:95. 10.3389/fcimb.2019.00095 31058096 PMC6482253

[29] Rosenwasser S Ziv C Creveld SG Vardi A. Virocell metabolism: metabolic innovations during host–virus interactions in the Ocean. Trends Microbiol. (2016) 24:821–32. 10.1016/j.tim.2016.06.006 27395772

[30] Xue Q Liu H Zhu Z Yang F Song Y Li Z. African swine fever virus regulates host energy and amino acid metabolism to promote viral replication. J Virol. (2022) 96:e0191921. 10.1128/JVI.01919-21 34908441 PMC8865428

[31] Delgado T Sanchez EL Camarda R Lagunoff M. Global metabolic profiling of infection by an oncogenic virus: KSHV induces and requires lipogenesis for survival of latent infection. PLoS Pathog. (2012) 8:e1002866. 10.1371/journal.ppat.1002866 22916018 PMC3420960

[32] Sanchez EL Carroll PA Thalhofer AB Lagunoff M. Latent KSHV infected endothelial cells are glutamine addicted and require glutaminolysis for survival. PLoS Pathog. (2015) 11:e1005052. 10.1371/journal.ppat.1005052 26197457 PMC4510438

[33] Thai M Graham NA Braas D Nehil M Komisopoulou E Kurdistani SK. Adenovirus E4ORF1-induced MYC activation promotes host cell anabolic glucose metabolism and virus replication. Cell Metab. (2014) 19:694–701. 10.1016/j.cmet.2014.03.009 24703700 PMC4294542

[34] Gou H Zhao M Yuan J Xu H Ding H Chen J. Metabolic profiles in cell lines infected with classical swine fever virus. Front Microbiol. (2017) 8:691. 10.3389/fmicb.2017.00691 28473819 PMC5397473

[35] Dixon SJ Lemberg KM Lamprecht MR Skouta R Zaitsev EM Gleason CE. Ferroptosis: an iron-dependent form of nonapoptotic cell death. Cell. (2012) 149:1060–72. 10.1016/j.cell.2012.03.042 22632970 PMC3367386

[36] Xu T Ding W Ji X Ao X Liu Y Yu W. Molecular mechanisms of ferroptosis and its role in cancer therapy. J Cell Mol Med. (2019) 23:4900–12. 10.1111/jcmm.14511 31232522 PMC6653007

[37] Zhang R Lei J Chen L Wang Y Yang G Yin Z. Gamma-glutamylcysteine exerts neuroprotection effects against cerebral ischemia/reperfusion injury through inhibiting lipid peroxidation and ferroptosis. Antioxidants. (2022) 11:1653. 10.3390/antiox11091653 36139727 PMC9495808

